# Disclosing medical errors: how do we prepare our students?

**DOI:** 10.1186/s12909-023-04125-3

**Published:** 2023-03-28

**Authors:** Dirkie Swinfen, Mathys Labuschagne, Gina Joubert

**Affiliations:** 1 Clinical Simulation and Skills Unit, Faculty of Health Sciences, University of the Free State, 205 Nelson Mandela Drive, 9300 Bloemfontein, South Africa; 2 Department of Biostatistics, School of Biomedical Sciences, Faculty of Health Sciences, University of the Free State, Bloemfontein, South Africa

**Keywords:** Medical errors, Duty of candour, Disclosure of medical errors, Patient safety, Barriers to error disclosure, Doctor-patient communication skills training, Medical ethics, Litigation, Role modelling in medical education

## Abstract

**Purpose:**

Despite patient safety initiatives, medical errors remain common and devastating. Disclosing errors is not only ethical, but also promotes restoration of the doctor-patient relationship. However, studies show active avoidance of error disclosure and the need for explicit training. In the South African setting, sparse information exists in terms of undergraduate medical training in error disclosure. To address this knowledge gap, the training of error disclosure in an undergraduate medical programme was examined, against the background of the available literature. The objective was to formulate a strategy to improve error disclosure teaching and practice, with the goal of improving patient care.

**Methods:**

Firstly, the literature was reviewed regarding the training of medical error disclosure. Secondly, the undergraduate medical training in error disclosure was probed, by looking at the pertinent findings from a broader study on undergraduate communication skills training. The design of the study was descriptive and cross-sectional. Anonymous questionnaires were distributed to all fourth- and fifth-year undergraduate medical students. Data were predominantly analysed quantitatively. Open-ended questions were analysed qualitatively using grounded theory coding.

**Results:**

Out of 132 fifth-year medical students, 106 participated (response rate 80.3%), while 65 out of 120 fourth-year students participated (response rate 54.2%). Of these participants, 48 fourth-year students (73.9%) and 64 fifth-year students (60.4%) reported infrequent teaching in the disclosure of medical errors. Almost half of the fourth-year students (49.2%) considered themselves novices in error disclosure, while 53.3% of fifth-year students rated their ability as average. According to 37/63 (58.7%) fourth-year students and 51/100 (51.0%) fifth-year students, senior doctors seldom or never modelled patient-centred care in the clinical training setting. These results resonated with the findings of other studies that showed lack of patient-centredness, as well as insufficient training in error disclosure, with resultant low confidence in this skill.

**Conclusion:**

The study findings confirmed a dire need for more frequent experiential training in the disclosure of medical errors, in undergraduate medical education. Medical educators should view errors as learning opportunities to improve patient care and model error disclosure in the clinical learning environment.

**Supplementary Information:**

The online version contains supplementary material available at 10.1186/s12909-023-04125-3.

## Introduction

Medical error has been defined as “a preventable adverse effect of medical care” [1]. Medical errors can have devastating consequences including death, disability, skyrocketing healthcare costs and erosion of society’s trust in healthcare [2]. Healthcare workers contributing to an error may become “the second victim” due to the effects of guilt and self-reproach [3]. To combat the detrimental effects of medical errors, there has been a worldwide drive to improve patient safety [2, 4]. The South African Department of Health issued a national guideline for patient safety incident reporting and learning in the public health sector of South Africa in 2017 [5].

Despite these measures, the axiom “to err is human” rings true and medical mistakes remain common [2]. Researchers consider medical errors the third leading cause of death in the USA [6]. An estimated 134 million hospital-based adverse events occur in low- to middle-income countries worldwide, contributing to approximately 2.6 million deaths annually [4]. In public hospitals in the Gauteng Province of South Africa, healthcare workers were linked with 6 910 serious detrimental incidents in 2021, including 1 954 deaths. [7]. In the South African public healthcare setting, factors such as staff shortages and the sheer numbers of patients with a high burden of disease, increase the risk of medical errors [8–10]. Nationwide information regarding the rate of errors in South Africa is limited [11]. The high incidence of medical malpractice claims and the large sums for medical litigation claims paid out by the South African government suggest a high frequency of medical errors, but could also be due to a greater awareness of patients’ rights in a society increasingly aware of their legal rights [9, 10].

In view of the inevitability and frequency of errors, healthcare practitioners should not only endeavour to avoid errors, but also be able to manage mistakes ethically [11]. This includes the reporting of errors, taking precautions to prevent recurrence and disclosure of errors to patients or their next of kin. The General Medical Council, the medical regulator in the United Kingdom, has outlined the obligation to disclose errors in a guideline titled “the duty of candour” [12]. This duty entails admitting that the treatment did not go to plan and explaining the short- and long-term consequences clearly. If indicated, an apology should be extended to the patient and a remedy or support offered [12]. Guidance from the South African medical regulator, the Health Professions Council of South Africa (HPCSA), emphasises that doctors have a moral obligation to be truthful and act in the best interest of the patient [13]. Disclosing the error is not only the right thing to do, but evidence has shown that “disclosure promotes closure” for patients or their families and doctors alike. The restoration of the therapeutic relationship is then more likely and the possibility of legal action is reduced [14, 15].

Despite these noble and practical motivations to be candid, doctors find the reporting and disclosure of errors awkward and painful and might be tempted to “sweep mistakes under the rug” [16]. A substantial gap has been observed between doctors’ intentions to disclose errors and their actual practices [17]. In a study of disclosure behaviour, it transpired that 98.5% of participating doctors described an error made, but only 11% of them had disclosed the error to the patient or their families [18].

A South African ophthalmologist and medical educator who found that many doctors did not use the incident reporting system at an academic hospital, investigated barriers to disclosure of medical errors. Barriers to the reporting of medical errors and the disclosure thereof to patients included lack of senior support, fear of being stigmatised by colleagues and fear of legal action [10]. He concluded that “Training for doctors in correct methods for adequate disclosure and apology will assist improving patient care” [10]. Error disclosure has been embraced as part of the core communication skills curriculum of undergraduate medical training in the UK and the USA [10, 19].

In the South African setting, doctors and postgraduate trainees reported ineptitude in error disclosure and rudimentary training in complex communication skills, especially in postgraduate training [10, 20]. However, questions emerged regarding the state of training in error disclosure at undergraduate level, how confident medical students are in this complex skill and how training in error disclosure can be improved.

To answer these questions, findings on the undergraduate medical training in error disclosure was examined. These formed part of a broader study on the undergraduate training in doctor-patient communication as a whole, at the University of the Free State [21]. The objective of this paper is to present the specific findings regarding training in medical error disclosure, against the background of the relevant literature, in order to formulate strategies to improve the disclosure of medical errors to patients. The goal is to improve patient care.

## Methods

### Study setting and design

A literature review was done to examine the ethical requirements in terms of error disclosure, to identify barriers to error disclosure and examine error disclosure behaviour. Medical education literature was probed to find best practice guidance in terms of training medical students to master this complex skill. The findings of the literature review have been incorporated into the introduction and discussion sections of the article.

A subsection of a study examining communication skills training in an undergraduate medical programme was analysed [21]. This subsection focused on training in error disclosure and student self-ratings of this skill. The study was conducted in Bloemfontein, South Africa, at the medical school of the University of the Free State (UFS) in June 2019. The study design was mainly quantitative, descriptive, and cross-sectional.

### Participants, questionnaire and data collection

The minimum duration of the MBChB course at the UFS is five years. The initial phase of six months is followed by a pre-clinical phase of two years. After a six-month introduction to the clinical phase, the two full years of clinical training take place in the fourth and fifth years of study. For the study, all undergraduate medical students in their fourth and fifth years of study at the UFS were asked to take part, thus no sampling was done. Junior students were not included in the study as they could not review the clinical phase of the training.

The researchers followed the scholarly guidelines of Botma and co-workers [22] to develop the questionnaire. A literature study was done to identify the key concepts in doctor-patient communication skills training. The use of national and international literature contributed to the validity of the questionnaire and guided the development of the questionnaire.

The questionnaire (see Appendix 1), based on Harden’s extended vision of the undergraduate medical curriculum, had the following themes: outcomes, content, educational methods, assessment methods, learning opportunities and educational environment relating to the training of doctor-patient communication [21, 23]. Questions were mostly quantitative and focused on the frequency of training and training opportunities. To enhance reliability, questions were placed in a logical order and kept free of jargon. The questionnaire contained statements rather than questions, so that respondents could agree or disagree by choosing an option on a modified Likert scale. The categories “often” and “almost always” were grouped as occurring frequently when referring to the results in the text, while “not at all” and “seldom” were grouped as occurring infrequently. The preferred educational method had to be ranked as 1, while the number 6 signified the least favoured method. Participants were given the opportunity to motivate their answers and add additional comments.

To enhance reliability a pilot study was done on ten randomly selected students, five fourth year and five fifth-year students. The pilot study indicated that the questionnaire did not require adjusting prior to conducting the study, except for isolated typing errors. The data of the pilot study have been included in the results.

Data collection was done through printed anonymous questionnaires. These were distributed to all fourth and fifth year undergraduate medical students during the orientation session at the start of their respective clinical rotations.

### Analyses of data

Data from the questionnaires were entered into a Microsoft Excel sheet and quantitative analysis was performed by the UFS Department of Biostatistics. Categorical variables were summarised by frequencies and percentages, and numerical variables by medians and ranges. For the qualitative analysis, the first author reviewed answers to open-ended questions. Grounded theory was used to enable analysis of data. After reading the data, open coding was used to assign codes to excerpts of data with the same central idea. Thereafter axial coding was used to look for connections between the codes and to place them into categories. The categories were verified through consensus meetings with one of the co-authors. Selective coding was used to find the overarching theme emerging from the data. An example of anaylsis of an open question using grounded theory can be seen in Appendix 2. Direct quotes from students’ comments were indicated by their study year and questionnaire number in brackets after the quote; for example, [5.18] represented questionnaire number 18, completed by a fifth-year student.

### Ethical considerations

All research procedures in this study were conducted according to the relevant guidelines and regulations of the Declaration of Helsinki. The UFS Health Sciences Research Ethics Committee (HSREC) approved the study (ethics number UFS-HSD2019/0327/2506) and UFS Gatekeepers gave permission for the execution of the study. An information sheet explained that voluntary completion of the questionnaire implied informed consent for anonymous participation in the study. The information leaflet also included a statement that the research findings would be published. The allocation of a number to each completed questionnaire prevented disclosure of personal information.

## Results

Out of 132 fifth-year students, 106 participated, resulting in a response rate of 80.3%. Sixty-five out of 120 fourth-year students participated, yielding a response rate of 54.2%. Eight different language groups were represented by the participating students, with Afrikaans (33.3% of fourth year students, 57.5% of fifth year students), English (40.4% of fourth year students, 14.9% of fifth year students) and Sesotho (8.8% of fourth year students, 11.7% of fifth year students) being the most common.

### Tuition in the disclosure of medical errors

Regarding tuition in medical error disclosure, 48 fourth-year and 64 fifth-year students (73.9% and 60.4%, respectively) recounted infrequent training in the clinical phase of their training. Of the fourth year students, 26.1% reported frequent tuition in error disclosure, while 39.1% of fifth-year students described frequent tuition in this skill. These responses are summarised in Table 1.

### Students’ self-ratings of their ability to explain medical errors

Students were asked to rate their ability to explain medical errors. In terms of the final-year students, 17.1% rated themselves as excellent, 53.3% rated themselves as average at this skill, and 29.5% rated themselves as novices. Of the fourth-year students, 7.7% of students rated themselves as excellent, 43.1% rated themselves as average and 49.2% rated themselves as novices (see Table 2). Students were not required to motivate their self-ratings. In the fourth year group the largest percentage of Afrikaans and English students rated themselves as average whereas the majority of Sesotho speaking students and those of all other languages rated themselves as novice. In the fifth year group the largest percentage of Sesotho speaking students considered themselves as novice whereas in all other language groups the largest percentage indicated average.

**Table 1:** Students’ responses regarding training in the disclosure of medical errors

Taught to explain medical errors	4th -year students (n = 65)				5th -year students Phase I (*n* = 90**); Phase II (*n* = 91**); Phase III (*n* = 105**)			
	Not at all	Seldom	Often	Almost always	Not at all	Seldom	Often	Almost always
	*n* (%)	*n* (%)	*n* (%)	*n* (%)	*n* (%)	*n* (%)	*n* (%)	*n* (%)
Phase I*	37 (56.9)	24 (36.9)	3 (4.6)	1 (1.5)	47 (52.2)	26 (28.9)	13 (14.4)	4 (4.4)
Phase II*	20 (30.8)	33 (50.8)	11 (16.9)	1 (1.5)	30 (33.0)	37 (40.7)	21(23.1)	3 (3.3)
Phase III*	12 (18.5)	36 (55.4)	14 (21.5)	3 (4.6)	20(19.0)	44 (41.9)	30 (28.6)	11 (10.5)

*Phase I: introductory phase (first 6 months); Phase II: pre-clinical phase (next 2 years); Phase III: clinical phase (final 2½ years).

** Some fifth year students only joined the programme after Phase II and only respond regarding Phase III.

**Table 2:** Students’ self-rating of ability to explain medical errors and apologise if required

Self-rating of ability to explain medical errors	4th -year students (*n* = 65)			5th -year students (*n* = 105*)		
	Novice	Average	Excellent	Novice	Average	Excellent
	*n* (%)	*n* (%)	*n* (%)	*n* (%)	*n* (%)	*n* (%)
Total group	32 (49.2)	28 (43.1)	5 (7.7)	31 (29.5)	56 (53.3)	18 (17.1)
Language group**						
Afrikaans	8 (42.1)	9 (47.4)	2 (10.5)	12 (22.2)	34 (63.3)	8 (14.8)
English	9 (39.1)	12 (52.2)	2 (8.7)	5 (35.7)	6 (42.9)	3 (21.4)
Sesotho	3 (60.0)	1 (20.0)	1 (20.0)	5 (45.4)	4 (36.4)	2 (18.2)
Other	8 (8.0)	2 (20.0)	0 (0)	5 (33.3)	7 (46.7)	3 (20.0)

*One fifth-year student did not complete this questionnaire item. ** Eight fourth-year students and 12 fifth-year students did not indicate their language

### Students’ preferences in terms of educational methods used

Students were asked to rate the usefulness of the various educational methods used to develop skills in communicating with patients. Both year groups ranked small group practice with simulated patients as the most useful method: “*Simulated patients create a more hands-on experience*” [5.54]. The fifth-year students gave observation with feedback after a real consultation an equally high rating: “*Doctor can help you immediately to correct your errors*” [5.4]. Both student groups considered lectures and video recordings of student consultations the least useful methods to teach communication skills. One student commented that “*skills cannot be solely acquired through passive observation*” [5.45].

### Educational environment

As part of probing the educational environment, students were asked whether senior doctors modelled patient-centred communication during clinical rotations. This question polarised opinions, with 58.7% of fourth-year and 51.0% of fifth-year students indicating that senior doctors infrequently modelled patient-centred communication, while 41.4% of fourth-year students and 49% of fifth-year students reported that senior doctors regularly modelled patient-centred behaviour. Students expressed their concern regarding the failure of doctors to talk to patients, the lack of empathy and the predominant emphasis on the disease rather than the person. Descriptions of doctors’ communication with patients included “*unapproachable, dismissive*, *abrupt*” and even “*inhumane*”. A student observed that “*Doctors let their egos get in the way of proper doctor-patient communication. Humility goes a long way*” [5.30]. One student remarked that “*senior doctors talk over patients and not to patients*” [5.57]. Students suggested factors contributing to the poor communication: “*Heavy workload & low staffing allow little or no time for a relationship with the patient*” [4.23].

Conversely, students who described doctors as good role models in terms of patient-centred communication made the following comments: “*They are very professional and patients always seem contented with their handling*” [4.27], and “*There was a lot of emphasis on patient-centred communication during clinical rotations*” [5.1].

## Discussion

Both groups of undergraduate medical students reported infrequent training in the disclosure of medical errors. Although the global medical education literature contained student appraisal of error disclosure training interventions [24], no South African study could be found that specifically asked undergraduate students to assess undergraduate training in error disclosure. However, Moodley et al. [20] surveyed South African neurologists and neurology registrars. They reported an insufficient emphasis on the acquisition of complex interpersonal skills during undergraduate and postgraduate training.

The majority of fifth-year students rated their ability to disclose medical errors as average, while most fourth-year students rated themselves as novices in terms of error disclosure. A possible explanation for the higher self-rating among fifth-year students might be that they became more confident due to more time spent in the clinical environment. When home languages of students were taken into account, it emerged that Afrikaans and English speaking students gave themselves higher ratings than Sesotho speaking students, but the reason for this difference in self-rating was not clear and needs further investigation.

The lack of confidence in the ability to disclose errors, was also found in the aforementioned study of Moodley et al. [20]. Among the neurology registrars participating, 28.6% deemed themselves competent in error disclosure, 38.1% reported that they lacked competence, with the remainder unsure. Among the neurologists, less than half (48%) deemed themselves competent in error disclosure, 17.3% declared that they were not competent in this skill and 34.5% were unsure of their competence. In contrast, when asked about their confidence in terms of discussing life and death issues, 64.3% of registrars and 78.1% of neurologists considered themselves competent, with only 11.9% of registrars and 11.5% of neurologists reported a lack in competence in this communication skill. It thus seems that these doctors found it easier to discuss death than disclosing a medical mistake. Furthermore, in their capacity as medical teachers of undergraduate students, doctors might find it difficult to inculcate the art of error disclosure if they themselves are lacking in this skill.

Given the current state of undergraduate medical training of error disclosure, as well as the perceived confidence in this skill, the question remains how training in error disclosure and the practice thereof can be improved.

More than half of students in each year group reported that senior doctors seldom modelled patient-centred communication, with examples cited of doctors speaking about patients, rather than to them. This speaks of a culture where patient-centredness is not paramount. In the South African medical literature, Carmichael comments on the potential effects of role modelling on error disclosure behaviour: “Positive role models have been reported to be important in training junior doctors but negative role models are even more impactful in entrenching a culture of ‘burying errors” [10].

This sentiment is echoed by Kling, who explored the culture of medicine in South Africa in terms of managing errors [11]. She concluded that there appears to be a tendency to cover up mistakes. She points to the myth of medical infallibility, which leads to impossible expectations and in the case of an error, shame and concealment. She acknowledges the need for sufficient staff training in error disclosure and a change in the medical culture. This need for training in error disclosure is also voiced by Carmichael, who surveyed doctors at the Witwatersrand medical school [10]. Of the 211 doctors surveyed, 94% agreed that training in error disclosure “is necessary to improve skills and facilitate effective disclosure” [10].

Worldwide research shows that doctors tend to avoid error disclosure, as it goes against the inclination for self-protection [14, 16, 25]. Detsky aptly describes this phenomenon as ‘Ethics says yes, but instinct says no’. It is therefore crucial that error disclosure is explicitly included in communication curricula and not left to chance. Medical educators have increasingly included error disclosure in communication curricula globally [10, 19]. However, from the findings of this study and a perusal of the relevant literature, South Africa has yet to follow suit.

So how can error disclosure training be improved in the South African undergraduate medical programme? We propose a four-fold strategy, based on the study findings and the salient aspects of the literature. Firstly, it would be helpful if the South African medical regulator could issue clear ethical guidelines regarding the disclosure of errors. The current ethical guidance states the duty to be honest [13], but there is no specific guidance regarding error disclosure. An example of a clear guideline is the Duty of Candour outlined by the medical regulator in the United Kingdom [12], but that, of course, needs to be contextualized to the South African setting.

Secondly, there should be greater institutional support when medical errors are made. Openness should be rewarded rather than punished. If there is a blame culture, the culture of rationalization and concealment of errors will be allowed to fester. Kling comments on the so-called ‘apology laws’ that exist in at least 34 states of the United States [11]. These provide legal protection for doctors who disclose mistakes and might reduce fear of litigation. However, the South African legal system is beyond the remit of this article.

Thirdly, staff development could help doctors and particularly medical teachers to undergo a paradigm shift in their approach to medical errors. Rather than seeing mistakes as ‘shameful secrets’, errors could be seen as opportunities for learning and growth. Staff development can help medical teachers become aware of their influence as role models and the importance of a more patient-centred approach [26]. As Kling clearly stated: “Respect for persons dictates that full disclosure be made to the patient or family” [11].

Fourthly, training in error disclosure should become a key part of the communication skills curriculum in medical training. Evidence shows that experiential learning such as role play with simulated patients are effective in nurturing complex communication skills. In studies conducted, students reported that didactic teaching combined with practical learning increased their confidence in doctor-patient communication [24, 27]. This was mirrored in the findings of this study, where students expressed a clear preference for active learning and learning in the clinical environment.

### Strengths and limitations

Strengths of the study were the high response rate, as well as the diversity of participants. The data collection tool allowed the probing of multiple aspects of doctor-patient communication training in a limited amount of time. Students were able to express their opinions and motivate their answers through the open-ended questions. The fact that the questionnaire was anonymous, enabled students to reflect honestly on sensitive topics such as negative role modelling in the clinical setting.

The cross-sectional design limited the conclusions that can drawn, as it only provided “a snapshot in time”. The fact that the study only examined the training from the students’ perspective, means that the picture is incomplete, as the perspectives of medical educators and patients are not represented. Recall bias might have affected the accuracy of participant responses.

### Recommendations and practical implications of the research

The disclosure of medical errors should form part of the formal content of doctor-patient communication skills training in undergraduate medical programme. Evidence for effective training of complex communication skills suggests using a combination of didactic and experiential methods. A stepwise approach, resembling that of conveying bad news, can be used [10].

The example set by practicing doctors is essential: “*Modelling of appropriate disclosure by attending physicians is paramount to avoid the blame-shifting, minimizing, and rationalizing of errors that will likely be emulated by trainees*” [25].

Further research is required to establish the knowledge, attitudes and behaviour of clinical teachers regarding the disclosure of medical errors in the South African medical education setting.

## Conclusion

Participants in this study reported infrequent training in error disclosure and inconsistent role modelling of patient-centred communication by medical teachers. The study findings were scrutinized in the light of the applicable global and South African literature to propose a four-fold strategy for improving the practice and training of error disclosure. Clearer guidelines from the medical regulator will mandate and aid error disclosure. At a local level, there should be institutional support for error disclosure. Staff development for medical teachers might help to change the culture of concealment and provide the required skills to practice and teach error disclosure. The training in error disclosure should form an explicit part of the undergraduate medical curriculum and should not be left to chance.

However, we must not be naive like Boxer in Orwell’s Animal Farm, with a blinkered outlook and a fix-it-all credo of “I will work harder” [28]. Working harder to promote a culture of openness and teaching the disclosure of medical errors will contribute to the improvement of patient care, but underlying system failings that make healthcare unsafe, such as insufficient staff or inadequate resources, also need to be addressed.

Disclosing a medical error can eventually help to mitigate the remorse and anguish associated with the error, known as second-victim trauma, which can lead to doctors considering leaving the profession [27]. It is true that “*the doctor who makes the mistake needs help too*” [3, 29]. In summary, although disclosing an error remains challenging, it can help restore trust in the medical profession. As medical educators, we need to make ourselves vulnerable and accountable, showing our students that mistakes are “*teachable moments in doing the right thing*” [25].

## Electronic supplementary material

Below is the link to the electronic supplementary material.

Supplementary Material 1

Supplementary Material 2

## Data Availability

The data that support the findings of this study are available from the corresponding author upon reasonable request.

## Declarations

### Disclaimer

The views expressed in this article are those of the authors and not of the University of the Free State or HWSETA.
