# Supplementary material for: Disclosing medical errors: how do we prepare our students?
Source: BMC Med Educ. 2023 Mar 28;23:191. doi: 10.1186/s12909-023-04125-3 (PMC10054053; doi:10.1186/s12909-023-04125-3)
Supplement: Supplementary file 1 — Supplementary Material 1 [file 12909_2023_4125_MOESM1_ESM.docx]

APPENDIX 1

The sections relevant to this paper have been highlighted.

FINAL QUESTIONNAIRE FOR STUDY TO OBTAIN A STUDENT REVIEW OF UNDERGRADUATE DOCTOR-PATIENT COMMUNICATION SKILLS TRAINING AT THE UFS

You have been asked to participate in a research study. Please note that by completing this questionnaire you are voluntarily agreeing to participate in this research study. You will remain anonymous and your data will be treated confidentially at all times. You may withdraw from this study at any given moment during the completion of the questionnaire. The results of the study may be published.

**Biographic data**

| i. How old are you? | years | |  |
| --- | --- | --- | --- |
| ii. What is your gender? |  | |  |
| iii. In which year of study are you? | 4^th^-year | 5^th^-year |  |
| iv. What is your home language? |  | |  |
| v. In which phase did you start training at UFS? | Phase I  Semester 1 | Phase II  Semesters  2–5 | Phase 3III  Semesters  6–10 |

If you were not present during a certain phase of training, please indicate with n/a (not applicable)

**Question 1: OUTCOMES**

- 1. The outcomes of doctor-patient communication skills training were made clear to us. Indicate your answers with an "x" in the relevant column.

|  | Not at all | Seldom | Often | Almost always |
| --- | --- | --- | --- | --- |
| Phase I |  |  |  |  |
| Phase II |  |  |  |  |
| Phase III |  |  |  |  |

1.2 We were told about the roles of the health practitioner as expected by the Health Professions Council of South Africa, such as Health Professional, Communicator and Collaborator.

|  | Not at all | Seldom | Often | Almost always |
| --- | --- | --- | --- | --- |
| Phase I |  |  |  |  |
| Phase II |  |  |  |  |
| Phase III |  |  |  |  |

**Question 2: CONTENT**

2.1 Educators told us about the studies that demonstrated the benefits of good communication skills for patient outcomes.

|  | Not at all | Seldom | Often | Almost always |
| --- | --- | --- | --- | --- |
| Phase I |  |  |  |  |
| Phase II |  |  |  |  |
| Phase III |  |  |  |  |

2.2.1 We received training in taking a thorough patient history, including a psychosocial history.

|  | No training | Limited training | Extensive training | Extensive training & practice |
| --- | --- | --- | --- | --- |
| Phase I |  |  |  |  |
| Phase II |  |  |  |  |
| Phase III |  |  |  |  |

2.2.2 How would you rate your ability to take a thorough history?

| Novice | Average | Excellent |
| --- | --- | --- |
|  |  |  |

Motivate your answer:

……………………………………………………………………………………………

……………………………………………………………………………………………

……………………………………………………………………………………………

2.3.1 Doctor-patient communication skills training included practical sessions in "breaking bad news"

|  | No training | Limited training | Frequent training | Extensive training & practice |
| --- | --- | --- | --- | --- |
| Phase I |  |  |  |  |
| Phase II |  |  |  |  |
| Phase III |  |  |  |  |

2.3.2 How would you rate your ability to break bad news to a patient?

| Novice | Average | Excellent |
| --- | --- | --- |
|  |  |  |

Motivate your answer:

……………………………………………………………………………………………………………………………………………………………………………………………………………………………………………………………………………………….

2.4.1 Doctor-patient communication skills training included learning how to manage language and cultural differences in the consultation.

|  | Not at all | Seldom | Often | Almost always |
| --- | --- | --- | --- | --- |
| Phase I |  |  |  |  |
| Phase II |  |  |  |  |
| Phase III |  |  |  |  |

2.4.2 How would you rate your ability to manage language and cultural differences in the consultation?

| Novice | Average | Excellent |
| --- | --- | --- |
|  |  |  |

Motivate your answer

……………………………………………………………………………………………………………………………………………………………………………………………………………………………………………………………………………………….

2.5.1 Doctor-patient communication skills training helped me to understand the patient's point of view.

|  | Not at all | Seldom | Often | Almost always |
| --- | --- | --- | --- | --- |
| Phase I |  |  |  |  |
| Phase II |  |  |  |  |
| Phase III |  |  |  |  |

2.5.2 How would you rate your ability to see the patient's point of view?

| Novice | Average | Excellent |
| --- | --- | --- |
|  |  |  |

2.6.1 Doctor-patient communication skills training involved explaining medical errors and potentially offering an apology to patients.

|  | Not at all | Seldom | Often | Almost always |
| --- | --- | --- | --- | --- |
| Phase I |  |  |  |  |
| Phase II |  |  |  |  |
| Phase III |  |  |  |  |

2.6.2 How would you rate your ability to explain medical errors and potentially offering an apology to a patient?

| Novice | Average | Excellent |
| --- | --- | --- |
|  |  |  |

2.7.1 The content of the doctor-patient communication skills training included learning how to defuse anger.

|  | Not at all | Seldom | Often | Almost always |
| --- | --- | --- | --- | --- |
| Phase I |  |  |  |  |
| Phase II |  |  |  |  |
| Phase III |  |  |  |  |

2.7.2 How would you rate your ability to defuse anger?

| Novice | Average | Excellent |
| --- | --- | --- |
|  |  |  |

**Question 3: EDUCATIONAL METHODS**

3.1 Which of the following methods of doctor-patient communication skills training did you find the most useful? Please rank the following methods, with **1 being your preferred method and 6 the least preferred method.**

If the method was not used, indicate N/A for not applicable.

Indicate during which phase or phases of training the method was used.

| **Teaching Methods** | **Ranking**  **(1 to 6)** | **Indicate phase(s) in which the method was used (I, II or III)**  **(Can be more than one phase)** | | |
| --- | --- | --- | --- | --- |
|  |  | **I** | **II** | **II** |
| 1. Lectures |  |  |  |  |
| 1. Small group practice with peers |  |  |  |  |
| 1. Small group practice with simulated patients |  |  |  |  |
| 1. Looking at videos of consultations. (Examples of good clinical communication and/or poor clinical communication) |  |  |  |  |
| 1. Being observed by a doctor or other healthcare professional while communicating with a patient in a ward or clinic & receiving feedback about your communication skills. |  |  |  |  |
| 1. Video-recording of student consultation for evaluation of doctor-patient communication skills. |  |  |  |  |

Explain the reason for choosing your preferred method in Question 3.1:

……………………………………………………………………………………………………………………………………………………………………………………………………………………………………………………………………………………….

Explain the reason for choosing your least preferred method:

……………………………………………………………………………………………………………………………………………………………………………………………………………………………………………………………………………………….

3.2 The simulated patients that we came encountered in practice sessions and OSCE situations were believable and made me feel like I was talking to a real patient.

| Never | Sometimes | Often | Almost always |
| --- | --- | --- | --- |
|  |  |  |  |

Motivate you answer: ……………………………………………………………………………………………………………………………………………………………………………………………………………………………………………………………………………………….

**Question 4: LEARNING OPPORTUNITIES**

4.1 We received enough opportunities to practise doctor-patient communication skills.

|  | Not at all | Seldom | Often | Almost always |
| --- | --- | --- | --- | --- |
| Phase I |  |  |  |  |
| Phase II |  |  |  |  |
| Phase III |  |  |  |  |

4.2 We have been given the opportunities to reflect on situations when our doctor-patient communication did not go well and how we could have managed it better.

|  | Not at all | Seldom | Often | Almost always |
| --- | --- | --- | --- | --- |
| Phase I |  |  |  |  |
| Phase II |  |  |  |  |
| Phase III |  |  |  |  |

**Question 5: ASSESSMENT**

5.1 The content of assessments, such as tests, demonstrated to me that doctor-patient communication skills are viewed as important.

|  | Not at all | Seldom | Often | Almost always |
| --- | --- | --- | --- | --- |
| Phase I |  |  |  |  |
| Phase II |  |  |  |  |
| Phase III |  |  |  |  |

5.2 The content of examinations demonstrated to me that doctor-patient communication skills are viewed as important.

|  | Not at all | Seldom | Often | Almost always |
| --- | --- | --- | --- | --- |
| Phase I |  |  |  |  |
| Phase II |  |  |  |  |
| Phase III |  |  |  |  |

5.3 Doctor-patient communication skills of students have been assessed continuously so that it can reflect everyday behaviour and attitudes.

|  | Not at all | Seldom | Often | Almost always |
| --- | --- | --- | --- | --- |
| Phase I |  |  |  |  |
| Phase II |  |  |  |  |
| Phase III |  |  |  |  |

5.4 What type of assessment is the best way to test communication skills in your opinion?

…………………………………………………………………………………………….

Motivate your answer.

……………………………………………………………………………………………………………………………………………………………………………………………………………………………………………………………………………………….

**Question 6: LEARNING ENVIRONMENT**

6.1 The learning environment enhances the development of good doctor-patient communication skills.

|  | Not at all | Seldom | Often | Almost always |
| --- | --- | --- | --- | --- |
| Phase I |  |  |  |  |
| Phase II |  |  |  |  |
| Phase III |  |  |  |  |

Motivate your answer

……………………………………………………………………………………………………………………………………………………………………………………………………………………………………………………………………………………….

6.2 During our clinical rotations, patient-centred communication is modelled by senior doctors.

| Never | Sometimes | Often | Almost always |
| --- | --- | --- | --- |
|  |  |  |  |

Motivate your answer: ……………………………………………………………………………………………………………………………………………………………………………………………………………………………………………………………………………………….

7. Doctor-patient communication skills cannot really be learnt: A person is either a natural communicator or not.

| Strongly disagree | Disagree | Agree | Strongly agree |
| --- | --- | --- | --- |
|  |  |  |  |

8. Are there any aspects of the doctor-patient communication skills training that you have found especially useful? Please motivate your answer.

……………………………………………………………………………………………………………………………………………………………………………………………………………………………………………………………………………………….

9. Are there any aspects of the doctor-patient communication skills training that you did not find particularly helpful? Please motivate your answer.

……………………………………………………………………………………………………………………………………………………………………………………………………………………………………………………………………………………….

10. Are there any further recommendations or comments you would like to make regarding the doctor-patient communication skills training?

……………………………………………………………………………………………………………………………………………………………………………………………………………………………………………………………………………………….……………………………………………………………………………………………………………………………………………………………………………………………………………………………………………………………………………………….

Thank you for your contribution to this research.
