# Supplementary material for: Disclosing medical errors: how do we prepare our students?
Source: BMC Med Educ. 2023 Mar 28;23:191. doi: 10.1186/s12909-023-04125-3 (PMC10054053; doi:10.1186/s12909-023-04125-3)
Supplement: Supplementary file 2 — Supplementary Material 2 [file 12909_2023_4125_MOESM2_ESM.docx]

**Appendix 2**

Example of using grounded theory for evaluating students' response to the open-ended component of Question 6.2. in the questionnaire.

Grounded theory coding has been used to interpret responses regarding the witnessing of patient-centred communication by senior doctors.

| **Open coding** | **Axial coding** | **Selective coding** |
| --- | --- | --- |
| Doctors focused on disease rather than concerns of patients | Not patient-centred | Due to a variety of external and internal factors, patient-centred care is not consistently modelled in the clinical environment, with unprofessional behaviour frequently mentioned. |
| Doctors focused on trainees and not speaking to patients |  |  |
| Variability of communication: some excellent examples seen and contrasted with poor examples | Inconsistent role-modelling |  |
| Students were shocked by doctors' demeaning and unprofessional behaviour. | Unprofessional behaviour |  |
| Time pressure has a negative influence on communication | Environmental factors |  |
| Work overload has an impact on communication with patients |  |  |
